# Emerging Perspectives on Leukemia Inhibitory Factor and its Receptor in Cancer

**DOI:** 10.3389/fonc.2021.693724

**Published:** 2021-07-29

**Authors:** Joe Christianson, Julia Thom Oxford, Cheryl L. Jorcyk

**Affiliations:** ^1^Department of Biological Sciences, Boise State University, Boise, ID, United States; ^2^Biomolecular Sciences Program, Boise State University, Boise, ID, United States

**Keywords:** LIF, LIFR, tumor progression, IL-6 cytokine family, metastasis, cancer stem cell, interleukin like EMT inducer, hippo signaling pathway

## Abstract

Tumorigenesis and metastasis have deep connections to inflammation and inflammatory cytokines, but the mechanisms underlying these relationships are poorly understood. Leukemia Inhibitory Factor (LIF) and its receptor (LIFR), part of the interleukin-6 (IL-6) cytokine family, make up one such ill-defined piece of the puzzle connecting inflammation to cancer. Although other members of the IL-6 family have been shown to be involved in the metastasis of multiple types of cancer, the role of LIF and LIFR has been challenging to determine. Described by others in the past as enigmatic and paradoxical, LIF and LIFR are expressed in a diverse array of cells in the body, and the narrative surrounding them in cancer-related processes has been vague, and at times even contradictory. Despite this, recent insights into their functional roles in cancer have highlighted interesting patterns that may allude to a broader understanding of LIF and LIFR within tumor growth and metastasis. This review will discuss in depth the signaling pathways activated by LIF and LIFR specifically in the context of cancer–the purpose being to summarize recent literature concerning the downstream effects of LIF/LIFR signaling in a variety of cancer-related circumstances in an effort to begin teasing out the intricate web of contradictions that have made this pair so challenging to define.

## Introduction

The interleukin-6 (IL-6) family cytokine LIF was originally discovered as an inducer of differentiation and inhibitor of proliferation in a murine myeloid leukemia cell line, where it originally received its name (1). However, LIF has since been demonstrated to be expressed by a variety of different cell lines with diverse downstream effects. The most well-known function of LIF is its role in maintaining murine embryonic stem cells (mESC) in culture by maintaining their totipotency and enhancing their self-renewal (2), an effect that is not seen in human ESCs. LIF has essential activities outside of ESC self-renewal and has been demonstrated to play an important role in mediating interactions between the embryo and the maternal environment. During development, LIF signaling is necessary for human blastocyst implantation (3) through mediating the invasiveness of trophoblastic cells (4). As such, LIF may represent a target for non-hormonal contraception (5), and has been suggested as a potential biomarker for the success of *in vitro* fertilization (6). Additionally, LIF expression is important in suppressing the maternal immune response during embryological implantation (3).

**Figure 1:**
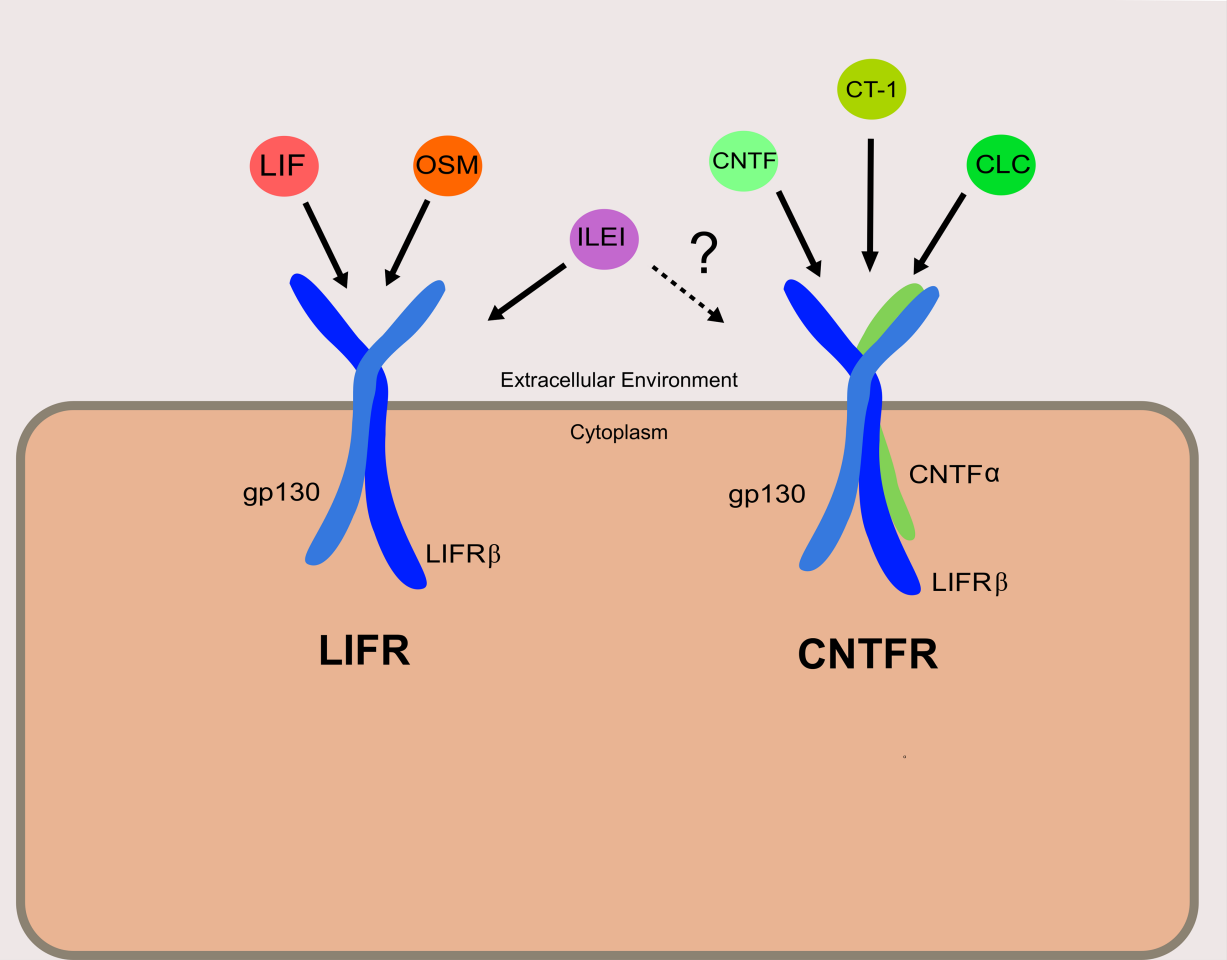
Receptor complexes and ligands utilizing LIFRβ. The left depicts the LIFR complex, and right depicts the CNTFR complex. Ligands are shown associated with their designated receptor complexes. It is confirmed that ILEI can bind to and elicit signaling through LIFR, though it is not clear if ILEI can signal through the CNTFR as well.

Discovered shortly after the ligand for which it is named, LIFRβ is a subunit of both the LIFR and the ciliary neurotrophic factor receptor (CNTFR) (**Figure 1**). The LIFR is a heterodimer consisting of LIFRβ and glycoprotein 130 (gp130), while CNTFR is a trimer of LIFRβ and gp130 with an additional CNTF-α receptor subunit. LIF is only one of a whole host of cytokines known to bind to LIFRβ (**Figure 1**). These include ligands that are part of the interleukin-6 (IL-6) cytokine family: namely, oncostatin M (OSM), ciliary neurotrophic factor (CNTF), cardiotrophin-1 (CT-1), and cardiotrophin-like cytokine (CLC) (7). Recently, interleukin-like EMT inducer (ILEI) was determined to be a ligand of LIFRβ, though further studies will be necessary to determine the precise receptor complex that ILEI utilizes (8).

It is apparent that LIFRβ plays a significant role in post early embryological development stages, as indicated by the rare congenital disease Stüve-Wiedemann Syndrome (SWS), which is caused by a truncated LIFRβ subunit. SWS is characterized by skeletal deformities, cardiac and respiratory distress, temperature dysregulation, and mild cognitive impairment (9, 10). To what degree these symptoms are due to impaired LIF signaling is challenging to determine, though, as LIFRβ has other ligands as previously mentioned. To further illustrate this point, LIFRβ knockouts in mice, while not embryonically lethal, result in premature death shortly after birth—likely due to significant neural, metabolic, bone, and placental defects (11). LIF knockouts on the other hand are not lethal, implying the potential for functional redundancy among ligands for LIFRβ with regards to development.

It is this very concept of functional redundancy, in addition to the fact the LIF and LIFRβ exhibit such clear polyfunctionality, that make the pair so obscure within the context of our understanding of cancer. The following will discuss LIF-LIFR signaling from a general perspective, and then transition to a more precise conversation regarding these signaling pathways within cancer. In addition, the review hopes to also touch on how our perspectives of LIF-LIFR signaling have grown more nuanced—with the addition of signaling pathways such as the Hippo pathway, the possible overlap with other LIFR ligands, the mechanisms through which LIF and LIFR have been classified as either pro-tumor growth/metastasis, or tumor growth/metastasis suppressive.

## LIF-LIFR Signaling

**Figure 2:**
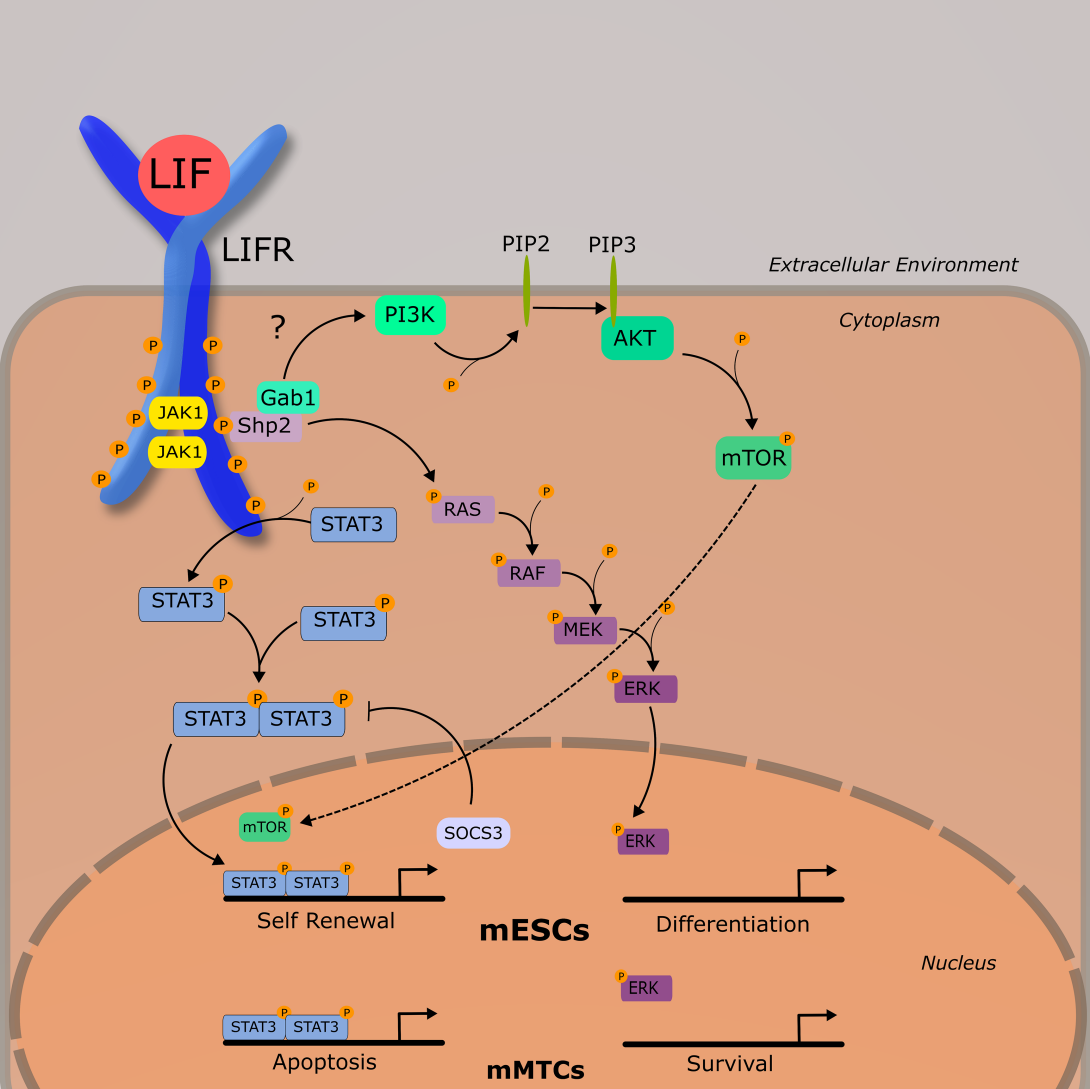
LIF-LIFR Signaling Network Schematic demonstrating the three primary signaling pathways activated by LIF-LIFR interaction: JAK/STAT3, MAPK, and PI3K/AKT. Murine embryonic stem cells (mESCs) and murine mammary tissue cells (mMTCs) have apparently different downstream effects of LIF signaling, demonstrating the pleiotropic nature of this cytokine.

The LIFR complex is a heterodimer consisting of gp130 and LIFRβ. Intracellularly, the LIFRβ/gp130 receptor complex famously signals through the JAK/STAT pathway and is constitutively associated with a janus associated kinase (JAK) family member—JAK1, JAK2, and TYK3 (12). The most demonstrably important is JAK1, as various knockout models for JAK1 exhibit significantly dampened responses to LIF as well as other IL-6 cytokines (13). Unlike other IL-6 family members, LIF has a high affinity for both gp130 and LIFRβ, and it is hypothesized that an ordered binding process is unlikely (14). Once bound to either subunit, LIF induces receptor heterodimerization, leading to the activation of a JAK1. Once activated, JAK1 phosphorylates tyrosine residues on both LIFRβ and gp130, which provide docking sites for various signal cascade components including signal transducer and activator of transcription 3 (STAT3) and the cytokine signaling inhibitor phosphatase SHP2. The activation of SHP2 by JAK1 is generally thought to be the mechanism through which the MAPK and PI3K pathways are activated, as SHP2 activation is required for the downstream phosphorylation of ERK1/2 (15). Although LIFR-mediated activation of PI3K/AKT pathway is less understood than others discussed in this review, it is generally accepted that SHP2, and perhaps GAB1, bind to the p85 subunit of PI3K in ESCs (16). This ultimately leads to the activation of the downstream transcriptional regulator mTOR. Of these three discussed pathways thus far (JAK/STATs, MAPK, and PI3K/AKT), JAK/STAT3 appears to be dominant, as STAT3 has 4 binding sites on both the LIFRβ and gp130, whereas SHP2 has one. As such, study of LIF and its receptor have been primarily focused on the JAK/STAT pathway. More information on the biochemical nature of this process can be found in an excellent review published by Nicola and Babon (7). Once phosphorylated, STAT3 forms a homodimer with another STAT3, and enters the nucleus where it acts as a transcription factor for various genes associated with increased proliferation and enhancing stem cell self-renewal, most notably Myc and Nanog (**Figure 2**) (17).

### Downstream LIFR Signaling and Crosstalk

Activation of STAT3 is followed by the rapid upregulation of the inhibitory protein, suppressor of cytokine signaling 3 (SOCS3). As the name implies, SOCS3 acts to inhibit the JAK/STAT pathway by binding to and leading to the ubiquitination of JAK1 and gp130, as well as competing with SHP2 for binding sites on the LIFRβ-gp130 heterodimer, inhibiting MAPK signaling (18, 19). Regulation of LIFR signaling, though, does not seem to be solely dependent on transcriptional activity.

Research into early developing mouse embryos suggest that the PI3K and JAK/STAT pathways exist in a tentative balance with MAPK, with the prior necessary for ESC self-renewal and survival, and the latter with differentiation (7, 20), (**Figure 2**). LIF-induced pluripotency is highly dependent on the activation of STAT3 (21, 22) and cells expressing a non-functional STAT3 and grown in the presence of LIF are induced to differentiate (22).

In contrast to mESCs where the JAK/STAT is acting as a suppressor of differentiation, in developing murine mammary tissue JAK/STAT acts as a pro-apoptotic signal, and MAPK as a pro-survival signal. During post lactational regression, elevated levels of LIF were shown to induce cellular apoptosis in a STAT3-dependent manner *via* lysosomal mediated cell death (23–25), and LIF-induced STAT3 also leads to an upregulation of OSM and the OSM receptor (26). During ductal elongation, LIF was demonstrated to signal primarily through ERK1/2 as a survival signal. This is relevant in that it shows outcomes of LIF signaling are markedly different across tissue types—a concept that will be developed further as we begin to discuss LIF in cancer.

## LIF And LIFRβ Expression in Cancer

As summarized in **Table 1**, LIF and LIFRβ expression are linked to a variety of human cancers, many of which are associated with both negative and positive prognostic outcomes. As a whole, it appears that LIF activation of both the JAK/STAT and PI3K/AKT pathways are associated with the promotion of tumor growth and metastasis. On the other hand, LIFRβ expression seems to be connected to the tumor suppressor pathway Hippo, and thereby is correlated with decreased tumor growth and metastasis.

**Table 1:** Cancers where LIF or LIFRβ are demonstrated to have an effect on human cancer cells *in vitro* and/or *in vivo*.

Cancer Type	Involvement	Pathway	Citations
***Tumor/Metastasis Promoting***			
Breast Cancer*	Proliferation, Invasion, Metastasis	PI3K/AKT, JAK/STAT, AKT/mTOR	(27–33)
**Chordoma**	CSC Renewal	N/A	(34)
**Choriocarcinoma**	Invasion, migration, suppressed proliferation	JAK/STAT, miR-141, miR-21	(35) (36),
**Colorectal Cancer**	Anti-apoptotic, induced by HIF2a	JAK/STAT Downregulation of p53	(37, 38)
**Glioblastoma**	CSC Renewal	JAK/STAT, TGF-β****upregulates LIF	(39–41)
**Glioma**	CSC Renewal	ZEB1 represses LIF	(42)
**Kidney cancer**	Proliferation	N/A	(27)
Melanoma*	CSC Renewal, migration	BMP, upregulation of stemness genes	(43–45)
**Nasopharyngeal Carcinoma**	Metastasis	SRC/YAP	(46)
**Oral Squamous Carcinoma**	Migration, invasion	Tumor cells recruit fibroblasts to release of LIF and TGF-β	(47)
**Osteosarcoma**	Growth/metastasis, CSC maintenance	JAK/STAT	(48, 49)
**Ovarian**	Survival, proliferation, metastasis	JAK/STAT	(50)
Pancreatic*	Tumor Growth, Activation of tumor associated fibroblasts	JAK/STAT	(51–54)
**Prostate**	Immunosuppressive, proliferation, castration resistance	JAK/STAT	(27, 55, 56)
**Rhabdomyosarcoma**	Migration	STAT3, AKT, MAPK	(57)
***Tumor/Metastasis Suppressive***			
Breast Cancer*	Metastasis Suppressor	Hippo, STAT3	(58–62)
**Cervical**	Growth Suppression	Suppression of HPV oncogenes	(63)
**Clear Renal Cell Carcinoma**	Metastasis Suppressor	Hippo, decreased YAP expression	(64)
**Gastric Cancer**	Growth arrest Invasion/metastasis suppressor	PI3K activity increases after LIFR downregulation	(65–67)
**Glioblastoma**	Invasion/Metastasis suppressor	PTEN/STAT3	(41)
**Hepatocellular Carcinoma**	Metastasis suppressor	PI3K attenuation	(68–70)
**Medullary Thyroid Cancer**	Growth Arrest	JAK/STAT	(71)
Melanoma*	Growth arrest, metastasis suppressor	TGF-β/STAT3/p21	(72)
Pancreatic*	Metastasis Suppressor	Increased E-Cadherin	(73)

This table is broken down into cancers where LIF and/or LIFRβ are involved in either the growth and metastasis of cancer, or the suppression of growth and metastasis. (*) indicates cancers that have both metastasis-promoting and-suppressing relationships with LIF and/or LIFRβ.

Despite conflicting evidence as to the precise role of LIF across cancer types, some interesting patterns have emerged, including the role of LIF in maintaining cancer stem cells (CSCs) in glioma, chordoma, melanoma, osteosarcoma, and glioblastoma (**Table 1**). Generally speaking, cancer stem cell maintenance by LIF and LIFR seem to follow a similar trend as that seen in mESCs: LIF signaling leads to the activation of STAT3, which increases the stem cell like properties in solid tumors through transcriptional regulators. LIF is not always the sole cause, though, as in ovarian cancer IL-6 and LIF work together to stimulate STAT3 phosphorylation and stemness, while the loss of either LIF or IL-6 highly abrogates this process (50). To add to this point, glioma initiating cells (which exhibit stem cell like qualities in glioma) are stimulated to produce LIF following signaling *via* TGF-β, leading to an increase in STAT3 phosphorylation (39).

Another pattern seen is the propensity for LIF signaling to result in migration and metastasis, something seen in its close relative OSM and IL-6 in multiple cancers, most notably, breast cancer (74, 75). In both instances, metastasis is highly dependent on the activity of STAT3, though other pathways such as MAPK, PI3K/AKT, and the Hippo pathway have also been linked to LIF/LIFRβ dependent effects on the oncogenic process. Aberrant JAK/STAT signaling has been linked to a variety of pathological states, including but not limited to various immune disorders such as rheumatoid arthritis, as well as cancers such as prostate and breast cancer (76, 77). Specifically, STAT3 overactivity has been associated with the invasion and proliferation of a significant variety of cancer cells both *in vitro* and *in vivo*, and as such has been recognized to be a strong oncogene.

But LIF does not seem to be solely dependent on STAT3 activation in order to be pro-oncogenic, and some have even pointed to tumor cell dormancy induction *via* a LIF : LIFR : STAT3 axis in breast cancer to bone marrow metastasis (62). For example, PI3K overactivation is commonly associated with the increased survival and proliferation of cancer cells. Activation of this pathway *via* LIF stimulation is correlated with apoptotic resistance in cholangiocarcinoma, but not with increased growth or metastasis (78). Furthermore, in the breast cancer cell lines MDA-MB-231 and T47D, treatment with, as well as transient overexpression of LIF led to increased mTOR activity and the phosphorylation of 4EBP1 and p7056K, which are downstream targets of this pathway and play roles in apoptotic resistance as well as protein synthesis (31). Overall, in these cell lines investigators found that LIF activity was correlated with increased growth *in vivo* and metastatic qualities *in vitro* (31). LIFR influences the PI3K/AKT pathway in a variety of cancers including prostate (79, 80), gastric (66), hepatocellular (70), nasopharyngeal (81) and rhabdomyosarcomas (57). In some instances, LIFRβ expression has been associated with decreased PI3K/AKT activity. Interestingly, while LIF has been demonstrated to exert effects on lipid metabolism in both the brain and in adipocytes *via* the PI3K/AKT pathway, little research has been done to evaluate how LIF signaling influences cancer metabolism in both glioblastoma and glioma, or in other cancer related pathological states.

### LIF-Induced Immunosuppression

The relationship between tumor cells and the immune system is a highly complex process, and extensive evidence suggests that many tumors actively suppress the host immune response as a way to prevent immune-mediated tumor destruction. LIF-induced immunosuppression has been recently demonstrated in prostate cancer cells (55) and glioblastoma (82).

LIF as an immunomodulator/suppressor in cancer represents an important potential target for treatment. In a study of glioblastoma, the presence of high levels of LIF in the tumor microenvironment (TME) was associated with an increased number of tumor-associated macrophages (TAMs). Higher levels of TAMs in the TME was shown to prevent the invasion of CD8+ T cells *via* the repression of CXCL9 secretion, thus hindering an effective immune response to cancerous tissue (82). To examine this phenomenon *in vivo*, glioblastoma patient xenograft models in immunocompromised mice were treated with a neutralizing monoclonal antibody (nAb) for LIF. Treatments with LIF nAb greatly reduced TAMs, as well as led to an increased accumulation of CD8+ T cells. Overall, the study found that high levels of LIF were associated with decreased response to anti-PD1 therapies, and that LIF nAb when used in conjunction with anti-PD1 therapy could be a potential therapeutic option for patients with solid tumors exhibiting high LIF expression (82). In 2019, a humanized LIF nAb called MSC-1 entered phase 1A clinical trials and has been recommended to enter phase 2 dose trials for patients with relapsing or non-responsive solid-state tumors (83). Clearly, LIF and the LIFRβ have relevant connections to cancer growth and metastasis that warrant additional research and definition.

### Tumor and Metastasis Suppression

In contrast to LIF typically being associated with the increased invasion and metastasis of cancer, LIFRβ expression has been shown to be correlated with the opposite. While LIFRβ is typically downregulated in a variety of cancers, it’s co-receptor gp130 is ubiquitously expressed in the human body, even detectable in serum, and it’s expression pattern across types of cancer is highly variable (84–86). In a variety of cancers, LIFRβ expression has been associated with higher patient survivability, and increased metastasis-free survival (**Table 1**), and that depletion of this receptor is somehow linked to decreased cellular adhesion and more aggressive cancer phenotypes through the inactivation of the Hippo pathway. We will discuss the Hippo pathway in further depth later on in this review.

Although LIFRβ signaling and its downstream targets have been well studied, how this receptor is regulated in cancer is poorly understood. Some have postulated that decreased LIFRβ expression occurs *via* an epigenetic mechanism such as LIFRβ promoter methylation, which has been observed in breast (87), clear renal cell carcinoma (64), hepatocellular carcinoma (68, 69), and colorectal cancer (88). Unfortunately, there is little research overall in regard to the mechanisms by which LIFRβ is regulated in cancer. Expression of LIFRβ is downregulated by miR-125a (58), miR-125b (89), and miR-9 (59) in a variety of human cancer cell lines. There is also some evidence pointing to hypoxia downregulating LIFRβ, as hypoxic conditions decreased LIFRβ expression in breast cancer cells and multiple hypoxic responsive elements have been identified in the LIFRβ promotor (62).That same group also identified histone deacetylase (HDAC) as a potential mechanism for LIFRβ downregulation, which is supported by evidence indicating that LIFRβ is upregulated when breast cancer cells were treated with HDAC inhibitors (28) (62). Notably, LIFRβ was upregulated in gastric cancer cells *in vitro* following transfection with the long non-coding RNA LNC-LOWEG, and this was correlated with decreased capacity for migration (65). In myeloid and placental cell lines, the LIFRβ gene was shown to be regulated by the transcription factor RUNX1, which has been shown to be important in leukemia, as well as breast cancer (90, 91).

While LIFRβ expression seems to be negatively correlated with breast cancer growth and metastasis (59, 60, 87), high expression of LIF is positively correlated (27, 30–32). The triple negative breast cancer cell line MDA-MB-231 was shown to highly express LIF, and treatment with LIF neutralizing antibodies impeded proliferation (27). On the other hand, overexpression of the LIFRβ in this same cell line resulted in decreased metastasis *in vivo*, with no effect on proliferation (59). However, it should be noted that others have found that MDA-MB-231 cells are unresponsive to LIF, and that this cell line had undetectable levels of LIFRβ expression (59, 62). This is an excellent example of the challenge in discerning the role of LIF and LIFRβ in cancer, as even in a single cell line their effects are debated. Conversely, in pancreatic cancer high LIF expression is correlated with lower metastasis free survival (54), whereas induction of LIFRβ expression in pancreatic cancer cell lines *in vitro* and *in vivo* decreased proliferation and migration, increased E-cadherin expression, and was associated with more favorable patient outcomes (73).

This begs the question: Why is decreased LIFRβ expression associated with worse outcomes, especially metastasis, when the majority of its downstream signaling pathways are classically described as oncogenic in nature? High expression of LIF could potentially lead to decreased expression of LIFRβ *via* internalization and degradation, as LIF binds to LIFRβ with a high affinity and an over 24-hour half-life until ligand/receptor disassociation, as demonstrated in kinetic studies (92), but this is purely speculation. Although the precise reasons may differ among cancers, few have made significant forays into the underlying molecular mechanisms by which LIFRβ plays a role as a metastasis and tumorigenic suppressor. The most relevant underlying molecular mechanisms demonstrated have defined connections to the tumor suppressor Hippo pathway, and links between LIFRβ and the Hippo pathway have been demonstrated in breast (59), clear renal cell carcinoma (64), and gastric cancer (93). Although LIF-LIFR signaling activates a variety of pathways associated with cancer progression such as JAK/STAT and MAPK, more potent and significant activators of these pathways already exist and are potential targets for treatment. This is not to say that LIF and LIFRβ are not relevant; however, but rather that the focus of the conversation surrounding them in cancer should be shifted towards how LIF and LIFR can be understood through the lens of tumor suppression and promotion *via* the less understood Hippo pathway. The potential therapeutic and physiological significance of the relationship between LIF/LIFRβ and the Hippo pathway thus necessitates speaking of their interaction in more depth.

## LIF-LIFR Activation of the Hippo Pathway

### The Hippo Pathway

**Figure 3:**
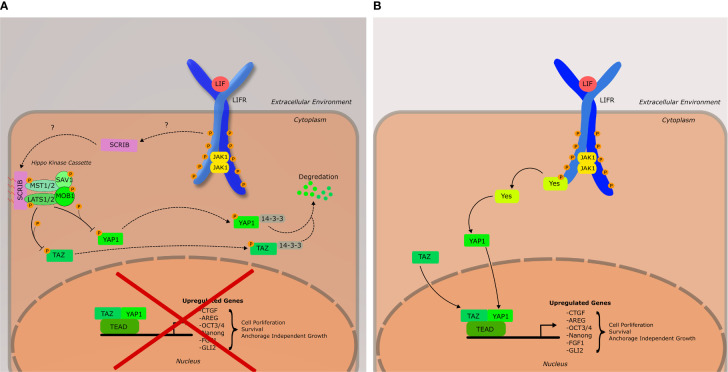
Schematic of the LIF-LIFR Mediated Hippo signaling network, as well as YES-YAP activation. **(A)** Hippo pathway is active, leading to the inhibition of YAP1 and TAZ. It should be noted that YAP1 and TAZ do not necessarily have to associate to have a downstream effect, nor is TEAD the only transcription factor they bind to. Furthermore, there are many upstream inputs that can activate the core Hippo kinase cascade that do not involve Scribble, or LIFRβ. Such inputs include cellular adhesion, metabolism, and cytoskeletal tension. **(B)** Hippo pathway is inactive; YAP1 and TAZ are active and aid in the regulation of genes associated with cell proliferation, survival, and anchorage independent growth. YAP1 has been shown to be activated by YES downstream of LIF-LIFR signaling.

The Hippo pathway, first discovered in Drosophila for its role in organ development, is a signaling cascade of particular interest to researchers due to its frequent dysregulation in human cancers (94). The primary effectors of this pathway are the transcription cofactors yes-associated protein (YAP) and transcriptional coactivator with PDZ binding motif (TAZ) (**Figure 3**). YAP and TAZ bind to a diverse array of transcription factors, the most important of which are members of the TEA Domain (TEAD) family. The Hippo pathway is activated by a variety of upstream cellular inputs, including various growth factors, cellular adhesion, and metabolic status resulting in the activation of salvador (SAV1). The core Hippo kinase cascade is as follows: SAV1 interacts with and activates MST1/2 (the mammalian homologue of the Hippo protein in *drosophila*) *via* an autophosphorylation event in the activation domain of MST1/2. Once activated, MST1/2 phosphorylates LATS1/2, leading to the recruitment of MOB1 to LATS1/2, whereupon MOB1 is also phosphorylated by MST1/2. The LATS/MOB1 complex is what engages and phosphorylates YAP (Ser127) and TAZ (Ser89). The phosphorylation of these serine residues generates binding sites for cytoplasmic 14-3-3 proteins, which sequester YAP and TAZ to the cytoplasm leading to their degradation. Dysregulation by increased expression or activation of YAP and TAZ have been found to be associated with malignant transformation and oncogenesis in numerous cancers, and thus their regulation (both at the transcriptional and protein levels) has become an area of importance in oncology, especially breast cancer (95–98). Thus, the Hippo pathway and its core kinases are tumor suppressors, while YAP and TAZ are oncogenes. The relationship between LIFRβ and the Hippo pathway is what initially defined LIFRβ as a metastasis suppressor in breast cancer (59).

### Mechanism of LIFR-Mediated Hippo Activation

The activation of the Hippo pathway *via* the LIFR was originally discovered by Chen et al. (59) in 2012 and their findings classified LIFRβ as a metastasis suppressor in cancer. A whole genome RNAi screen by Iorns et al. that same year (60) corroborated the conclusion of LIFRβ as a metastasis suppressor in breast cancer. Chen et al. demonstrated in a breast cancer model that LIFRβ expression is positively correlated with the membrane localization of the cell polarity protein Scribble to cadherins junctions, and resulted in decreased cellular migration and invasion which was dependent on the inactivation of YAP and TAZ (59). Interestingly, membrane localization of scribble was thought to require the expression of E-Cadherin, though the results of this study indicated otherwise.

Scribble is important in the maintenance of cellular polarity and has been demonstrated to have effects on the MAPK signaling pathway, as well as the Hippo pathway (95, 99). In the context of the Hippo pathway, scribble acts as a scaffolding protein for MST1/2 and LATS1/2 and TAZ (95). Upon localization to the cell membrane, this complex is active, and can begin the phosphorylation cascade that ultimately results in the cytoplasmic sequestration of YAP and/or TAZ *via* binding by 14-3-3 proteins (**Figure 3**).

In a recent study on clear renal cell carcinoma (CRCC), LIFRβ was found to be consistently downregulated in more aggressive cancers, likely due to promoter methylation and copy number variation (64). Silencing LIFRβ expression in CRCC cell lines led to an increase in the nuclear localization of YAP and enhanced migration and invasion. Most importantly, the silencing of YAP partially reversed this phenotype, indicating that loss of LIFRβ-promoted transformation is likely due to decreased Hippo activity, and therefore, increased transcriptional activity of YAP (64). The study in CRCC did not determine the mechanism by which LIFR activated the Hippo pathway, and in the years since Chen et al. originally established the Hippo-LIFR connection, the mechanism underlying the localization of scribble to the cell membrane *via* LIFR activation has not been determined. This is an important gap in the literature. Loss of cellular polarity is a hallmark of EMT, and if polarity-associated proteins such as Scribble are required for LIFR to activate the Hippo pathway then this gap could partially explain how LIF-LIFR signaling can have such a stark difference in downstream effect across disparate types of tissues and especially within cancer. But this is not the only association between LIF/LIFRβ and other effectors in the Hippo pathway. Interestingly, LIF activity has also been associated with the activation of YAP *via* the Src family kinase YES.

## LIFR Activation of YES: YAP Activity Downstream of LIF

### Background in ESCs

The connection with Src family kinases is a little researched facet of LIF/LIFR signaling. One such member of this family, YES, is a tyrosine kinase that activates YAP. The activation of YES by the LIFR has been shown to be relevant to LIF-induced stem cell maintenance, and unlike other pathways discussed thus far, seems to have little to no crosstalk with other LIF signaling pathways such as MAPK and JAK/STAT, at least in the context of mESCs (100). Research has shown that LIF-induced ESC self-renewal in mice is highly dependent upon LIF-mediated YAP-TEAD4 activation *via* YES, and these researchers determined that YES activity downstream of LIF had more profound effects on self-renewal than LIF-STAT3 (101). Although the precise mechanism is understudied, the proposed model is as follows. YES binds to the gp130 receptor subunit of the LIFR *via* an SH2 domain and is activated by JAK1. The active YES then then goes on to phosphorylate and activate YAP. YAP binds to and stimulates transcription with TEAD2, leading to the expression of the pluripotency factor OCT3/4 (101).

### Increased YAP Activity in Cancer

Recent studies on LIF in cancer have further demonstrated LIF-LIFR mediated YES activation. In a human *in vitro* pancreatic cancer model, LIF expression was highly correlated with increased YAP activity (54). In this instance, researchers were trying to understand the relationship between STAT3 and human KRAS driven pancreatic adenocarcinoma (PDAC), and this team hypothesized that LIF functioned in an autocrine manner, stimulating the growth of pancreatic cancer cells as well as their formation of 3D spheres in culture. They found that increased KRAS activity resulted in an increased expression of LIF. This effect was lost when downstream signaling proteins in the MAPK pathway were inhibited, suggesting that LIF upregulation in PDAC is dependent on MAPK activation. In general, LIF was found to be overexpressed in human pancreatic carcinomas relative to normal tissue, and that in a pan-cancer analysis LIF was significantly more upregulated in cancers with a mutation in KRAS. The silencing of LIF, though either genetic means or neutralizing antibodies resulted in an increased phosphorylation of YAP at ser127, and the activation of upstream Hippo pathway kinases (54). Furthermore, LIF nAbs used with gemcitabine significantly reduced the growth of patient-derived xenograft (PDX) tumors *in vivo.* These results are in stark contrast to earlier findings in breast cancer demonstrating that LIF-LIFR signaling activated the Hippo pathway, thereby inhibiting YAP and TAZ.

Additionally, a study of gastric cancer found that higher levels of LIF and LIFR were associated with increased proliferation, invasion, and metastasis (93). Interestingly they determined that LIF-LIFR signaling actually inhibited Scribble localization to cell membranes, thereby preventing the inactivation of YAP through the Hippo pathway. When YAP was inhibited *via* shRNA, the effect of LIF-LIFR signaling on cancer growth and migration was lost (93). In a dose dependent manner, LIF decreased the phosphorylation of MST and LATS, implying that LIF-LIFR signaling is somehow inhibiting the Hippo pathway and allowing YAP to remain active, rather than directly activating YES to activate YAP (93).

To further the complexity, in a model of nasopharyngeal carcinoma (NPC), cells constitutively expressing a cytoplasmic variant of LIF had a markedly lower expression of YAP as well as phosphorylated YAP at ser127, suggesting that although YAP expression was decreased, a higher proportion of YAP remained active relative to controls (46). Depletion of LIFRβ resulted in an increased expression of YAP, and a higher level of pYAP was also demonstrated—though, this could simply be due to the fact that more YAP was physically present in the cell. Clearly, though, this is showing another link between LIF/LIFRβ expression and YAP. There are further links between LIF/LIFRβ in YAP expression, as LIFRβ expression has been negatively correlated with YAP expression in clear renal cell carcinoma (64). In nasopharyngeal carcinoma (NPC) high levels of LIF are associated with higher degrees and radio resistance, tumor progression, and decreased DNA repair (81). Overall, the findings in both PDAC and NPC suggest that the relationship between LIF-LIFR signaling, YES-YAP activity and the Hippo pathway are significantly more nuanced than originally described in breast cancer models. Combined with the findings of LIF signaling leading to upstream inhibition of the Hippo pathway in gastric cancer, it is clear that LIF-LIFR-Hippo pathway interactions are highly tumor dependent. This should be unsurprising at this point, considering how this has been a recurring motif for LIF not only in cancer but physiologically as well. Looking across all cancers that LIF has been associated with, a closer examination of the Hippo pathway’s involvement in that cancer, if one has not been found, should be necessitated.

## ILEI: a Novel Ligand for LIFRβ

Interleukin-like EMT inducer (ILEI) is a cytokine-like protein of the FAM3C family that is speculated to have a four-helical bundle structure similar to LIF and has been implicated in a number of pathophysiological contexts, including Alzheimer’s and cancer metastasis (102). A recent study by Howe and colleagues (8) identified ILEI as a ligand for LIFRβ based upon a yeast 2-hybrid screen that was confirmed with crosslinking and immunoprecipitation experiments.

A series of experiments by Howe and colleagues sought to elucidate potential mechanisms by which TGF-β induced metastasis and CSC renewal in breast cancer, in which they found chronic stimulation of normal murine mammary gland (NMuMG) cells with TGF-β led to an increase in both LIFRβ and ILEI protein expression. Furthermore, they demonstrated that that ILEI activated STAT3 in a dose-dependent manner that was dependent on LIFRβ expression.

Immunocompromised mice injected with NMuMG cells expressing LIFRβ and ILEI had significantly higher host tumor burden and metastasis relative to controls, and this effect was partially lost in mice infected with cells with LIFRβ and ILEI knocked down. Intriguingly, in mice injected with NmuMG cells originally expressing LIFRβ, expression of LIFRβ was lost in sites of tumor outgrowth, as well as metastasis. This could be alluding to the role LIFRβ seems to play in tumor initiation, and CSC renewal, while simultaneously acting as a metastasis suppressor.

The induction of ILEI and LIFRβ expression by TGF-β is particularly interesting result, as TGF-β has been associated with the increased transcription of LIF in a number of cancers including in melanoma (72), thymic epithelium (103), glioblastoma (39), and in tumor associated stromal fibroblasts (104). Furthermore, there are some lines of evidence suggesting that TGF-β works in conjunction with the oncogenic transcriptional regulator c-Myc and OSM to cause the malignant transformation of human mammary epithelial cells (105–107). The relationship between OSM, TGF-β, and STAT3 implies there may be a crucial connection between the downstream effects of IL-6 cytokines and TGF-β.

## Looking Forward

### LIFR-HIPPO Activation *via* Alternative Ligands

As to date, published studies have only pursued a link between LIF/LIFR-mediated activation of the Hippo pathway, and one may be inclined to wonder if other LIFRβ ligands (especially OSM, considering both LIF and OSM can utilize the LIFR complex) also have the capacity to activate the Hippo pathway. Evidence of a role for other ligands is supported by the fact that transgenic mice who are LIF ^-/-^ (thought the LIFR is intact) exhibit only mild physiological deficits, whereas LIFRβ ^-/-^ die shortly after birth. Furthermore, in trophoblastic cell lines, it was shown that there is some degree of functional overlap between OSM and LIF in downstream effect (108). While hereditary LIFRβ mutations result in the rare developmental disease Stüve-Wiedemann syndrome, women who have a deficiency in LIF expression face the problem of infertility with little other apparent physiological differences. Therefore, if there is in fact LIFR activation of the Hippo pathway across multiple cell lines, it is highly likely that other ligands have the capacity to result in pathway activation, especially considering the Hippo pathway’s significant importance during development.

There is at least some tangential evidence of a relationship between other IL-6 cytokines and the Hippo pathway, especially YAP. In a murine heart failure model, YAP-TEAD activity was demonstrated to result in the upregulation of OSM and the OSMR and was directly associated with the dedifferentiation of cardiomyocytes. Interestingly, there was also a link between OSM activity, and a further upregulation of YAP, indicating there may be a potential positive feedback loop between OSM and YAP (109). In a murine model of breast cancer to bone metastasis, OSM was demonstrated to cause the upregulation and secretion of amphiregulin (AREG), a growth factor that lead to the differentiation of osteoclasts (75). Although the authors of this study did not elucidate the mechanism of AREG upregulation, separate studies have shown that YAP-TEAD activation in a human breast cancer model directly lead to an AREG increase (110), and similarly TAZ-TEAD induced migration and invasion of BC cells is partially abrogated when AREG is knocked down (111). Although this potential mechanism is purely speculative, this certainly begs the question as to whether or not OSM is modulating AREG expression through YAP, as there is already some evidence indicating that LIFRβ/gp130 complex has the capacity to activate YAP through the protein YES.

### Alternative LIF and LIFRβ Transcripts

On a final note, very few studies (both in cancer and in other fields) make a clear distinction between the intracellular and secreted forms of LIF. There have been three transcripts of the LIF gene identified in both mouse and human cells: LIF-T, LIF-M, and LIF-D (112, 113); to this point we have been almost exclusively discussing the secreted form LIF-D. Regulation of these transcripts is centered around the alternative transcription of the first exon, which contains the secretory signal sequence. While LIF-M can exist in the cytoplasm or can be secreted, LIF-T completely lacks the first exon containing the secretory sequence and is localized to the cytoplasm. Early research showed that alternative LIF transcripts had both a tissue-dependent expression profile, as well as unique functions, with the intracellular transcripts LIF-T and LIF-M demonstrated to initiate proapoptotic signaling independent of the LIFR (113, 114). There has been some recent data on these alternative transcripts, though, including a recent study of the African elephant which identified a LIF-M “like” protein participating in p53-mediated apoptosis. The African Elephant genome contains multiple copies of this LIF-M-like gene and was postulated by the authors to be a partial example of a solution to Peto’s paradox (115). Interestingly, high expression of an intracellular LIF mutant was associated with more invasive and aggressive tumors in nasopharyngeal carcinoma (46).

LIFRβ has an alternative structure as well — there is both a membrane bound and secreted form (116). Generally, it is hypothesized that soluble LIFRβ is that it is meant to bind up latent LIF in the extracellular matrix. As to what regulates this alternative transcript is unknown, though one could speculate that this is a response to a high LIF environment.

## Conclusion

The IL-6 family cytokine LIF and its receptor subunit LIFRβ have come to represent a challenge to understanding the role of inflammatory cytokines in cancer. Despite significant advances in our knowledge of how inflammation drives cancer progression and metastasis, LIF and LIFRβ provide particularly poignant demonstrations of how much there is to learn about the processes involved. There has been a significant focus throughout the years on STAT3 being the causal driver of LIF mediated effects in cancer, and not without cause—our primary understanding of LIF is derived through its effects on mESCs *via* STAT3. Other cytokines, specifically IL-6 and OSM, clearly have more profound effects in cancer through STAT3—this has left LIF in the proverbial wayside, as more potent activators of STAT3 have been targeted for study. Even the case for STAT3 being a driver of metastasis and tumor growth in breast cancer has been challenged, as there have been studies that have shown both LIF and OSM suppressing tumor growth and metastasis *via* STAT3 in breast cancer cell lines (62, 117, 118). In recent years, though, the apparent connection of LIF and specifically LIFRβ to the Hippo pathway have opened up a new avenue for our broadening understanding of how this cytokine functions. This has, in many ways, left us with more questions than answers: what could explain the data demonstrating that LIF activates YAP *via* the YES/gp130 pathway, while other studies show that LIFRβ inhibits YAP through the Scribble/Hippo pathway? Furthermore, is it possible for other ligands in the IL-6 family to activate these downstream signaling pathways as well? Indeed, all IL-6 family cytokines can bind to gp130, and many can bind to LIFRβ. These are just a small sample of many unanswered questions when it comes to LIF and LIFRβ in cancer, many of which are enticing avenues of research. With ILEI being a new ligand for LIFRβ and considering the development of a nAb against LIF in solid tumors in a clinical trial — there is a significant need in the field of immuno-oncology to more readily define the relationship to the Hippo pathway. Hopefully, this review will act as an aid to any researcher looking to further develop our emerging perspectives of LIF and its receptor in cancer.

## Author Contributions

Conceptualization: JC. Resources: CJ. Supervision: CJ. Writing (original), draft preparation: JC. Writing—review and editing: JC, JO, and CJ. All authors contributed to the article and approved the submitted version.

## Funding

METAvivor, M.J. Murdock Charitable Trust, Office of Research Infrastructure Programs, National Institutes of Health (P20GM103408, P20GM109095, R25GM123927, U54GM104944-06, 1C06RR020533).

## Conflict of Interest

The authors declare that the research was conducted in the absence of any commercial or financial relationships that could be construed as a potential conflict of interest.

## Publisher’s Note

All claims expressed in this article are solely those of the authors and do not necessarily represent those of their affiliated organizations, or those of the publisher, the editors and the reviewers. Any product that may be evaluated in this article, or claim that may be made by its manufacturer, is not guaranteed or endorsed by the publisher.

## Abbreviations

AREG, Amphiregulin; CLC, Cardiotrophin-Like Cytokine; CNTF, Ciliary Neurotrophic Factor; CSC, Cancer Stem Cell; CT-1, Cardiotrophin- 1; EMT, Epithelial to Mesenchymal Transition; E1KD, hnRNPE1 knockdown; gp130, glycoprotein 130; HDAC, histone deacetylase ; HMEC, human mammary epithelial cells, IL-6, interleukin-6; ILEA, Interleukin-Like EMT Inducer; JAK, Janus Associated Kinase; LIF, Leukemia Inhibitory Factor; LIFR, Leukemia Inhibitory Factor Receptor; mESC, Murine Embryonic Stem Cell; nAB, Neutralizing Antibody; NMuMG, Normal Murine Mammary Gland; NOD, Non-Obese Diabetic; NPC, Nasopharyngeal Carcinoma; NSG, NOD-SCID Gamma; OSM, Oncostatin M; PDAC, Pancreatic Ductal Adenocarcinoma; SCID, Severe Combined Immunodeficiency; SFK, SRC Family Kinase; SOCS3, Suppressor of Cytokine Signaling 3; SWS, Stüve-Wiedemann Syndrome; TAM, Tumor Associated Macrophage; TAZ, Transcriptional Coactivator with PDZ Binding Motif, TEAD, TEA-Domain; TGF-β, Transforming Growth Factor Beta; WT, Wild Type; YAP, Yes-Associated Protein.
